# Treatment of Hemorrhagic Malarial Fever

**Published:** 1874-10

**Authors:** E. D. McDaniel

**Affiliations:** Camden, Ala.


### TREATMENT OF HEMORRHAGIC MALARIAL FEVER

BY E. D. M'DANIEL, A.M., M.D., OF CAMDEN, ALA.

In the treatment the indications are,—

*First.* To stay the hemorrhage.

*Second.* To correct the derangements of the digestive function.

*Third.* To prevent additional paroxysms.

*Fourth.* To soothe the injured organs and to make suitable restitution for the blood that has been lost.

These Indications are to be met as follows:

*First.* Take about grs. xv. of calomel and grs. xv. of bicarbonate of soda, mix them well and divide into vi. equal powders, of which give one dry on the tongue, to be swallowed with a mouthful of cold water; give these powders continuously, one every two hours, until the constipation is overcome or the diarrhœa corrected. If any one of the powders is thrown back or believed to be thrown back by vomiting, let no time be lost, but another immediately given, and so on, until the vomiting is palliated, (and this will generally not be long) and let the insatiable thirst, in the meanwhile, be indulged with pellets of ice, if this can be had.

*Second.* Apply heat to the general cutaneous surface, either by the hot-air bath, or by the hot-vapor bath, or by surrounding the patient with hot irons, hot bricks, bottles of hot water, bags of hot salt, or otherwise as most eligible under the circumstances. A cataplasm of mustard should also be applied to the abdomen, and should extend high up on the chest so as to relieve the oppressed respiration as well as the nausea. The mustard should by no means be allowed to blister, but merely to redden. The feet and legs should be placed in a tub of strong mustard-water as hot as can be borne, and should be kept in this bath as long as can be borne, without decided fatigue. Not the least heated air or heated vapor should be allowed to be inhaled, but the face of the patient should be industriously fanned and wiped with ice or, in the absence of ice, with the coldest available fresh water. The cold wiping should be done rapidly and perseveringly but interruptedly, as by this method, its effects, which is exerted by stimulating reflex nerveaction—both local and general, both excito-motor and excito-vasor —and also by soothing and moderating the excessive and oppressive sensation of heat, may be most steadily and lastingly kept up. In this way the application of heat to the general surface may be almost indefinitely prolonged, and its brilliantly salutary effects—directly in restoring the skin functions, and indirectly in arresting the hemorrhage—will become promptly and conspicuously manifest. The sighing will cease; the nausea and vomiting will disappear; the jactitation and anxiety will subside, and the utterance of the patient instead of the despondent murmur, "Oh, I feel so bad," will be the grateful and hopeful admission, "I now feel better." But if, as will *exceptionlaly* happen, and that, too, when the surface of the body is pale, cold and collapsed—a condition of surface not at all incompatible, but on the other hand, often associated with a subjective sensation of most intolerable morbid heat—the hot applications can absolutely not be borne and should seem to augment the excessive existing debility and oppression, then they should be alternated by the old Russian dash, or replaced by the use of ice or cold water all over the body, according to the method already advised for the face. Now, I know that there are many honest and very intelligent physicians who regard the heat simply as an invariable stimulant and cold as necessarily an absolute sedative, and who will be at a loss to understand how two modes of treatments that are, superficially considered, so opposite as the two here designated, can be rationally consistent and both practically safe and successful. I may remind such that in a general sense, extremes may meet, and that the morbid effects of excessive heat and of excessive cold, as exhibited respectively in burns and in frostbites, are strikingly similar. And so in the therapeutical applications here advised, the heat and the cold ultimately reach the same curative result: the heat directly stimulates the cutaneous nerves and capillaries, and thus mainly acts in restoring to the skin the lost powers and functions of its nerves and exhalents: the cold imparts a shock—not one that is depressing but stimulating; one that excites reflex nerve-action and thus re-establishes vascular secretory functions that have been suspended. The fact that cold applied as here advised, is truly, really and practically a stimulant, is beyond all question. A bladder of pounded ice will, in these fevers, often produce the redness of a rosebud on a surface on which a sinapism will excite neither color nor sensation. I have, occasionally, merely by spring-water at 60° Fah. temporarily removed the icterode hue from the skin and revived temporarily the weakened capillary action. And that cold thus applied is not only a remedy occasionally allowable, but a well-established invaluable, heroic treatment in the congestive fevers of the tropics and of sub-tropical climates, and even in the congestive stage of such fevers, is a matter of accredited medical history, and is taught by Currie, Warren Stone, and so many other great masters of medicine, that it may be regarded as a canon in our medical creed.

That the restoration of cutaneous action, and especially of the perspiratory function, and the use of proper medicines of the purgative class, are the rational and appropriate remedies for the hemorrhage, which is the great and imminent danger in hæmaturic malarial fever, must, I think, be obvious from the pathology of the disease upon which I have insisted. That they are the remedies which are sanctioned by practical results is equally clear.

Dr. J. C. Osborne, of Greensboro, Alabama, doubly taught by observation and by experience, states that the warm bath gave more comfort and quiet than any other remedy. This testimony dates as far back as 1867. In the same year Dr. East, of Grimes county, Texas, treated cases of the disease, and afterwards published a paper in the *Galveston Medical Journal*, from which are taken the following extracts:

"I found the patient complaining of frequent chilliness, a hard, frequent jerking, though not full pulse, frequent discharges of bloody urine, with griping pain in the bowels; skin yellow, rough and dry. The tongue red on the margins, and coated with a yellow fur in the middle, bitter taste, conjunctiva deeply stained and vision clouded. The patient complained of epigastric uneasiness, spirits much depressed, languor and nervousness. There was a general discoloration of the skin, attended with smarting and itching. Bowels constipated. ℞. Mild chloride mercury, grs. x.; sacchar. alb., grs. xx. Make powders No. iij.; ordered one every three hours; also, a saturated solution of Epsom salts, teaspoonful every three hours; ordered, also, a hot pepper and mustard foot-bath under cover.

"On visiting the patient the next day, I found him quiet—had had three good discharges from the calomel and salts; free from fever; and had discharged no bloody urine since midnight. Ordered quinine, grs. iv., Dover's powder, grs. ij., every four hours, and also a teaspoonful of the salts solution every four hours, making a dose of one or the other every two hours.

"Was called to the patient again at 6 P.M. Symptoms all aggravated; began to get worse after the third dose of quinine and Dover's powder. Intense cephalalgia; suffusion of the eyes; debility; listlessness; dull countenance; discharging bloody urine every half hour; a most painful and persistent aching of the back; tongue loaded with a yellow fur, and breath very offensive. Ordered a hot salt and mustard foot-bath under cover, and directed gelsemin, grs. ss., spirits nit. æther, f. dr. j., every three hours in water.

"Next morning found patient better, passed no bloody urine after the second dose of gelsemin and nitre. Ordered the foot-bath at night for two or three nights, and the gelsemin and nitre to be kept up at intervals of four hours for two or three days, with a tablespoonful of the salts solution every morning. Ordered, also, a little egg-nog. Patient relieved."

Dr. East states that this same patient—John Stevens—had, a little more than one year afterwards, another attack of the same kind, in which substantially the same treatment was employed, with like happy effects from the purgatives and the hot applications, and the same deplorable symptoms rapidly following the use of quinine. He also states that about the same time he had three other kindred cases, and treated them similarly with similar results.

In a most valuable letter, kindly addressed to me by my highly-esteemed friend, Dr. A. J. Reese, and dated Mobile, Ala., April 2d, 1874, the author says: "I have concluded to write you a few lines on the subject of your essay, and particularly, to call your attention to the treatment which I found successful in Dallas county, in 1867, and subsequently. It is not my purpose to review the practice of any, but only to give the plan of treatment adopted by myself with almost universal success.

"First: A dose of calomel is given to commence its work of disgorging the mucous surfaces, removing the vitiated matter and assisting the liver to resume its functions so soon as reaction takes place. To produce this result—reaction—to restore an equilibrium in the circulation, and to force the skin to assist the over-burdened kidneys in performing double duty, I employ heat (one of Tyndall's modes of motion). For this purpose, a hot-air-bath, with patient well covered with blankets until there is free diaphoresis (the patient breathing pure air, as much as possible, in the meantime). When this point has been attained, and the vomiting ceases, the patient's thirst may be gratified with cold lemonade, which may be given as rapidly as absorbed. In the absence of lemons, I have used chlorate of potash as an oxygeneating agent. Should the artificial heat require assistance to stimulate the capillaries, atrophine, alone or combined with morphine, hypodermically, should be added. The hæmaturia ceases usually soon after reaction takes place, and there is free diaphoresis."

The same in tenor is the testimony of Dr. Robertson, of Birmingham, Ala., who says that he has had the greatest success by wrapping up his patients in blankets wrung out of hot water, and thus promoting perspiration.

Dr. J. Paul Jones, of Camden, Ala., tells me that about three years ago, he was sent for many miles to see a youth who was suffering from this pernicious form of fever, and found the patient in a state of a very considerable lapse. Being without any fixed opinions on either the pathology or treatment of such cases, he addressed himself to the relief of what seemed to him the most urgent symptom, namely, the collapse. He plied his patient with heat, by bricks, bottles, and poultices and the like, covered him with blankets and quilts, succeeded in getting reaction, and found that the hemorrhage ceased, and with it all the other very urgent symptoms. He has, ever since, uniformly adopted the same course of treatment in similar cases, and I know that he has inaugurated in his practice in such cases, a new era of most gratifying success.

Before I pass from this head of the treatment, I think it important to call your attention to some substitutes for calomel and soda, that seem to me worthy of careful trial with the view of correcting the conditions of the stomach, liver and bowels. Powdered ipecac, one or two grains, may be substituted for the two and a half grains soda with the calomel in the prescription above recommended; ipecac may also be used in place of the calomel, and linked with the soda for the same purpose; podophyllin has been highly recommended in small and repeated doses instead of the calomel, but I cannot guarantee that it is equally efficacious. I think it likely, on the theoretical grounds, that the carbonate of lithia—so efficient in lithic varieties of urinary deposits—would be better, associated with calomel, than is the soda. It deserves a trial here as well as in the prophylaxis to correct that condition of the blood and urine which leads to the nephritic lesion.

I also mention, in passing, that if you expect any benefit in this hæmaturia, from substances known to do good in the proper hemorrhaghic diathesis—such as alum, gallic acid, tannin, acetate of lead, and persulphate and perchloride of iron—your expectations will be disappointed, and for the reason that there is not here a charge of blood characterized by reduced fibrin and diminished coagulability, or by things abstracted; but on the other hand, a change dependent on things superadded, excrementitious matters being retained and constituting blood poisons and kidney irritants. The only hemostatic adjuvants worth one moment's consideration here, are arsenic, mur. tinct. of iron and hypodermic injections of fluid extract of ergot. I think well of these skillfully used. The arsenic, however, should never be given in the acute stage of the disease, in the nauseating form of Fowler's solution, but in powder with some demulcent substance, like powdered liquorice. The mur. tinct. iron cannot be given and retained until the condition of the stomach has been improved, and then it will be found that the hemorrhage has also, already ceased. These adjuvants will be rarely demanded.

3. I now proceed to the third indication of Treatment—the prevention of additional paroxysms. This is an easy task, if the previous indications have been successfully and efficiently met. The wonderful general impression made by our disease; the stunning shock to the nervous system; the fearful loss of blood; the grave, traumatic-like lesion inflicted on the kidney, and the other sudden great changes every where apparent, seem, temporarily, to break the morbid habit and to interfere, for a while, with the periodic tendency. If the gastric, intestinal and cutaneous functions be continually watched and properly sustained, I believe that the immediate demand for specific antiperiodics will be, by no means, great or imperative, and if, in any way temporarily contraindicated, their use may be, for a while, justifiably postponed. Now, my professional brethren, I know that I here step upon awful, solemn, dangerous and responsible ground; and being, in general, as you all know, an advocate of quinine to an extent that few prudent men can claim to exceed, I take the position that I do here, not without having made the proper, thoughtful, serious and conscientious pause. In Dr. East's experience, as far as I read it to you, and in much more that I did not read to you, just as often as he got his patients into a satisfactory condition and then commenced giving quinine, just so often *he plunged them back into hœmaturia,* with its unnumbered woes and dangers. And often, in conversation on this subject with physicians and others, I have had my attention called to cases in which hæmaturia followed quinine, apparently as effect does cause. And over and over, and over again, have I in my own practice observed the hæmaturia re-established or aggravated after quinine; and on the other hand, have seen the disease, with a very formidable array of symptoms, go handsomely on, without one grain of quinine, to a happy convalesence. Dr. A. J. Reese, in the letter already quoted, says on this point: "I wish to add, in conclusion, that quinine given in the congestive stage will hasten the hæmaturia. My attention was called to this fact in the first two cases I treated—males, ages respectively 26 and 45—the hæmaturia commencing in each a few hours after taking large doses of quinine—the discharges from the kidneys very copious. These two cases, together with all treated subsequently, were treated according to above plan—quinine withheld until the emunctories had resumed their work and hemorhagic action ceased."

Having placed before you this array of strong, indubitable and important facts, allow me to counsel you to farther and closer observation, and declining any advice as to whether or not you should give quinine in the bleeding stage of such cases, or very soon thereafter, to leave you entirely, for the present, to your own responsible judgment and discretion.

Where quinine produces obstinate emesis, it should not be given by the mouth at least, and emetics, if used at all, must be used with great caution throughout the treatment of the disease. The obvious effect of vomiting, and of all other straining efforts, is to augment the hemorrhage. But powdered white arsenic $\frac{1}{12}$ grain, with powdered liquorice root $\frac{1}{2}$ grain, is not nauseous, and being antiseptic, hemostatic and febrifuge, should not be omitted. It may be given every four or six hours.

4th. The fourth and last indication is to soothe the injured organs, and to replenish with blood the depleted vessels.

The kidneys should be sedulously guarded and protected from continued or additional irritation. Rest in the recumbent position must be secured; no irritating diuretic substances, such as cantharides, turpentine, or santonine, should be employed, either externally or internally; the urates must be antagonized by the use of the salts of lethia; demulcents and demulcent kidney-tonics, such as flax-seed tea with fluid extract buchu, may be combined with grateful, sweet spirits of nitre, and harmless diluents should be reasonably indulged. The stomach and bowels, irritated and tender, must be allowed repose; no solid food must be permitted, but the diet must be rigidly restricted to fluid or semi-fluid forms of light, bland and grateful aliments, until the digestive powers become gradually restored. Sweet milk alone, or with corn meal mush, if properly relished, will be excellent food. But in cases where the hemorrhage has been completely exhaustive, where the patient is constantly on the verge of deliquium, as where the least elevation of the head, or the least effort at self-movement, convulses his limbs with tremors, or veils his vision with darkness, or seals his pale lips in muteness, no hesitation should be allowed or time lost, but prompt recourse should be had to mediate or immediate transfusion. If human blood be not available, that of lambs or of calves may be defibrinated and used, or preferably to these, fresh cow's milk may be employed.

